# Synthesis, crystal structure and Hirshfeld surface analysis of hexa­aqua­nickel(II) bis­(4-hy­droxy­benzoate) dihydrate

**DOI:** 10.1107/S205698902200531X

**Published:** 2022-05-24

**Authors:** Abror Ruzmetov, Aziz Ibragimov, Jamshid Ashurov, Zebo Boltaeva, Bakhtiyar Ibragimov, Sultan Usmanov

**Affiliations:** a Institute of General and Inorganic Chemistry, Academy of Sciences of Uzbekistan, 100170, M. Ulugbek Str 77a, Tashkent, Uzbekistan; b Institute of Bioorganic Chemistry, Academy of Sciences of Uzbekistan, 100125, Kh. Abdullaev Str 83, Tashkent, Uzbekistan; c Institute of Chemical Sciences of Kazakhstan NAS, Walikhanov str. 106, Almaty, 050010, Kazakhstan; Universität Greifswald, Germany

**Keywords:** 4-hy­droxy­benzoic acid, Ni complex, X-ray crystallography, mol­ecular structure, hydrogen bonding

## Abstract

The title compound, [Ni(H_2_O)_6_](PHB)_2_·2H_2_O (**1**) (PHB = 4-hy­droxy­benzoate, C_7_H_5_O_3_), was obtained by the reaction of NiCl_2_, 4-hy­droxy­benzoic acid (PHBA) and mono­ethano­lamine in aqueous ethanol solution. The Ni^II^ ion is coordinated by six water mol­ecules and is located on an inversion center. The outer coordination sphere in the asymmetric unit comprises one PHB anion and one water mol­ecule, *i.e.* the compound is a salt and a hydrate consisting of three components. In the crystal, the components are packed into an inter­molecular network stabilized by O—H⋯O hydrogen bonds. A comprehensive Hirshfeld surface analysis was carried out to investigate the inter­molecular inter­actions in detail.

## Abstract 2

The title compound [Ni(H_2_O)_6_](PHB)_2_(H_2_O)_2_ (PHB = 4-hy­droxy­benzoate, C_7_H_5_O_3_), isostructural with the Mg, Co and Mn complexes, was obtained by the reaction of NiCl_2_, 4-hy­droxy­benzoic acid (PHBA) and mono­ethano­lamine in aqueous ethanol solution.

## 1. Chemical context

*Para*-hy­droxy­benzoic acid (PHBA) is a natural compound found in carrots, oil palm, grapes and others (Manuja *et al.*, 2013 ▸). It demonstrates a wide spectrum of biological actions including anti­microbial, anti­fungal, anti­algal, and anti­viral activity, the regulation of plant growth and other types of bioactivities (Manuja *et al.*, 2013 ▸; Cho *et al.*, 1998 ▸; Sytar *et al.*, 2012 ▸). As a result of the presence of carboxyl and hydroxyl groups, PHBA can easily form metal complexes (Lo *et al.*, 2020 ▸; Sekine *et al.*, 2018 ▸; Gomathi & Mu­thiah, 2013 ▸; Ibragimov *et al.*, 2017*a* ▸,*b* ▸). The biological properties of ligand compounds, *e.g.* benzoic acid derivatives, may be enhanced by metal complex formation (Tran *et al.*, 2020 ▸; Hassan *et al.*, 2020 ▸). The improvement of the biological action may be even more pronounced when an auxiliary ligand with the same bioactivity is inserted into the coordination sphere alongside the target ligand (Ibragimov *et al.*, 2017*c* ▸). Mono­ethano­lamine (MEA), which is found in a number of food items such as daikon radish, caraway, muscadine grape, *etc*. has noticeable anti­microbial (Zardini *et al.*, 2014 ▸), plant growth (Bergmann & Eckert, 1990 ▸) and other types of activities (Moussa *et al.*, 2019 ▸). It therefore appeared to be a suitable auxiliary ligand for the bioactivity enhancement of PHBA.

It can be anti­cipated that mixing a Brønsted base (MEA) with a Brønsted acid (PHBA) in a reaction medium also containing a metal salt (NiCl_2_) may lead to the formation of different types of compounds: (*a*) the desired mixed-ligand Ni complex with MEA in neutral and PHBA in carboxyl­ate forms; (*b*) both ligands coordinated in a neutral form with chlorine ions residing in the outer coordination sphere for compensation of the positive charge of the central nickel ion; (*c*) homoleptic complexes or those with only one organic ligand type plus water of coordination (and with or without anions in the outer coordination sphere for potentially needed charge compensation); or (*d*) a strictly organic salt between mono­ethano­lammonium (*i.e.* protonated amine) and *para*-hy­droxy­benzoate (*i.e.* deprotonated acid, PHB). However, we have obtained (*e*), a supra­molecular complex (**1**) based on the Ni^II^ ion with six coordinated water mol­ecules, two *para*-hy­droxy­benzoate anions in the outer coordination sphere and two lattice solvent water mol­ecules. 
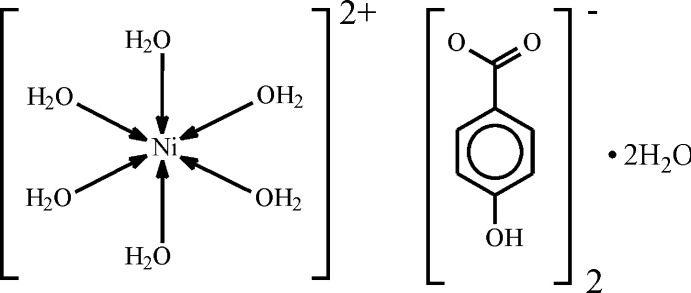


We presume that this structure is realized due to the energetic favorability of the obtained complex, in particular in the solid state, since the formation of the hexa­aqua­nickel(II) cation opens up the possibility of generating a multitude of stabilizing inter­molecular hydrogen bonds. The Brønsted acid–base reaction between the two mol­ecules intended as ligands apparently precedes complexation and/or crystallization and mono­ethano­lamine or its protonated cationic ammonium form are absent from the crystallized salt. Nearly half a century ago, complexes of magnesium(II), cobalt(II) and manganese(II), which are isostructural to the compound reported here, were obtained and structurally characterized by a group in Azerbaijan (Shnulin *et al.*, 1981 ▸, 1984 ▸). The accuracy of these structure determinations was low, although reasonable for that time. An analogous nickel(II) complex with *p*-nitro­benzoate counter-ions was recently obtained and published by us (Ibragimov *et al.*, 2018 ▸). Neither the inter­molecular inter­actions of this analogous complex salt nor those in the isostructural compounds have been estimated qu­anti­tatively as yet. Notably, despite a search of the CSD (Groom *et al.*, 2016 ▸) for the hexa­aqua­nickel(II) complex returning 352 hits, for only one of the reported crystal structures of [Ni(H_2_O)_6_]^2+^ salts was a Hirshfeld surface analysis carried out (Bednarchuk *et al.*, 2016 ▸). This left the cationic complex unconsidered and a corresponding analysis of [Ni(H_2_O)_6_]^2+^ is therefore unaccounted for to date. This communication is, hence, devoted to the crystal structure and comprehensive Hirshfeld surface analysis of the obtained supra­molecular complex salt **1**.

## 2. Structural commentary

**Figure 1:**
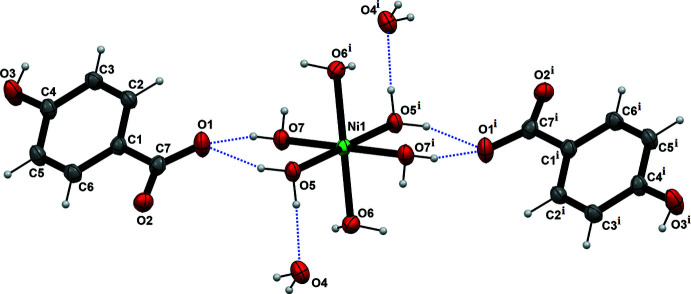
The mol­ecular structure of **1.** The ellipsoids of non-hydrogen atoms are drawn at the 50% probability level. Symmetry code: 1 − *x*, 1 − *y*, 1 − *z*.

The mol­ecular structure of **1** is shown in Fig. 1 ▸. The asymmetric unit of the structure consists of half of the nickel complex ion (residing on an inversion center), one *para*-hy­droxy­benzoate anion (PHB) and one water mol­ecule. The formula of the obtained compound is therefore [Ni(H_2_O)_6_](PHB)_2_·2H_2_O. The bond lengths between the metal center and the oxygen donor atoms of the water mol­ecules fall into the small range 2.0483 (13)–2.0893 (13) Å, while the bond angles vary between 88.72 (7) and 91.28 (7)°, *i.e.* the polyhedron around the central ion takes on the form of a nearly ideal octa­hedron. Compensation for the positive charge of the Ni^II^ ion is achieved with the deprotonation of PHBA mol­ecules during the course of the reaction resulting in the respective carboxyl­ate anions, which are incorporated in the outer coordination sphere. The carboxyl­ate group is nearly but not perfectly coplanar with the aromatic ring evidenced by the corresponding dihedral angle of 12.51 (3)°. The complex cations inter­act with the anions through the formation of O7—H7*B*⋯O1^vi^ [2.675 (2) Å] and O5—H5*B*⋯O1 [2.632 (2) Å] hydrogen bonds (Table 1 ▸) with an 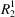 (6) graph-set notation (Etter *et al.*, 1990 ▸).

## 3. Supra­molecular features

**Figure 2:**
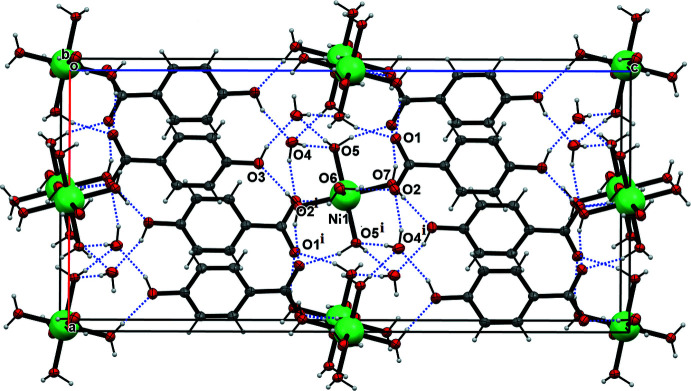
The packing of **1** viewed along the *b*-axis direction.

There are seven crystallographically independent oxygen atoms in the crystal structure, two of which serve only as hydrogen-bond acceptors (O1 and O2), three are both hydrogen-bond donors and acceptors (O3, O4, O5), and two are only hydrogen-bond donors (O6 and O7). All of the oxygen atoms are involved in relatively short inter­molecular hydrogen bonds between the [Ni(H_2_O)_6_]^2+^ cations, the PHB anions and the solvent water mol­ecules. The *D*⋯*A* distances of these bonds are in the range 2.632 (2)–2.785 (2) Å (Table 1 ▸), which is indicative of sufficiently strong inter­molecular inter­actions. The aromatic rings of the PHB anions are arranged in two different angles relative to the cell parameters and with an angle of 57.15° between their respective planes (Fig. 2 ▸). Adjacent anions with the same ring alignment adopt opposite orientations (alcohol and carboxyl­ate moieties on opposite sites of the mol­ecules alternate when viewed along the crystallographic *a*-axis). The complex cations are bridged by the length of the 4-hy­droxy­benzoate anions in the *c*-axis direction. The cations are linked in the *ab* plane by hydrogen bonds to water mol­ecules and the PHB alcohol and carboxyl­ate moieties. In consequence, layers of organic and inorganic sublattices alternate in the *c*-axis direction. Together, these inter­actions associate the components into a three-dimensional network (Fig. 2 ▸).

## 4. Database survey

A survey of the Cambridge Structural Database [Groom *et al.*, 2016 ▸; accessed January 2022 using *ConQuest* (Bruno *et al.*, 2002 ▸)] reveals that there are 352 hits in the database containing the hexa­aqua­nickel(II) complex ion. Nearly half a century ago, coordination complex formation with benzoic acid derivatives including PHBA was widely studied in the Azerbaijan Institute of Applied Physics. Researchers from this institute synthesized and structurally characterized supra­molecular complexes analogous to compound **1** with magnesium(II) (MGHBZA20; Shnulin *et al.*, 1981 ▸), cobalt(II) (MGHBZB20; Shnulin *et al.*, 1981 ▸) and manganese(II) (COLWUV; Shnulin *et al.*, 1984 ▸), which are all isostructural with the title compound. In addition, the structure of the magnesium(II) complex (AYOJOP; Baruah, 2016 ▸) is isomorphic with that of compound **1**. The precision of the previous structure determinations of these compounds were not nearly as high as that of the structure reported here (*R*-factors of 0.07 or more compared to 0.03) while the inter­molecular inter­actions have not yet been assessed qu­anti­tatively.

## 5. Hirshfeld surface analysis

**Figure 3:**
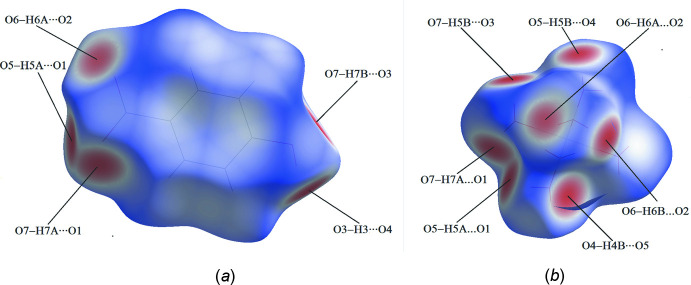
View of the three-dimensional Hirshfeld surfaces for (*a*) the PHB anion and (*b*) the [Ni(H_2_O)_6_]^2+^ cation of the title compound **1** plotted over *d*_norm_ in the range −0.4180 to 1.3344 a.u.

**Figure 4:**
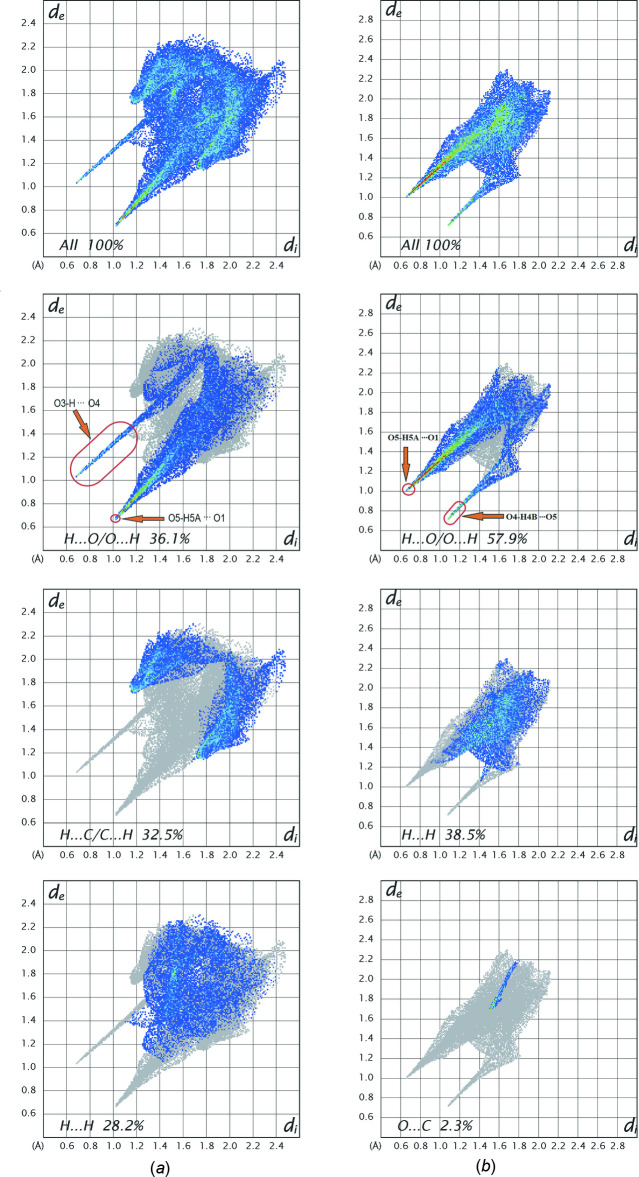
Two-dimensional fingerprint plots for (*a*) the PHB anion and (*b*) the [Ni(H_2_O)_6_]^2+^ cation.

Inter­molecular inter­actions can be assessed qu­anti­tatively by carrying out a Hirshfeld surface analysis (Spackman *et al.*, 2021 ▸). We have calculated Hirshfeld surfaces and fingerprint plots separately for the PHB anion and [Ni(H_2_O)_6_]^2+^ cation of compound **1**. The red spots on the surfaces show the pre­dom­inant strong inter­actions, which correspond to the O6—H6*A*⋯O2, O3—H3⋯O4, O5—H5*A*⋯O1, O7—H7*B*⋯O3, and O7—H7*A*⋯O1 hydrogen bonds, whereas the blue areas represent regions completely free from close contacts (Fig. 3 ▸). Despite the high mol­ecular symmetry of the complex cation, there are differences with regard to its Hirshfeld surfaces between the aqua ligands. Two aqua ligands (O5, O5*A*, *trans* to each other) are engaged in three contacts, while the others exhibit only two contacts. The *d*_norm_ surfaces of the title compound include hydrogen bonding with the solvent water mol­ecules, suggesting an increased stability of the hydrated form. The complete Hirshfeld surface analysis of the crystal structure shows that the major contribution to the inter­molecular inter­actions corresponds to strong H⋯O/O⋯H contacts. Fingerprint plots demonstrate that their contributions are 36.1% for PHB and 57.9% for [Ni(H_2_O)_6_]^2+^ (Fig. 4 ▸). Such a high percentage for the latter is unusual, but not unexpected considering that the complex ion contains six water mol­ecules coordinated to the nickel center. Next in overall significance are the H⋯H contacts, which contribute 28.2% and 38.5%, respectively, for the anionic and cationic fragments. However, in case of PHB, the contribution of H⋯H contacts is smaller than the H⋯C/C⋯H contribution (32.5%) whereas the latter inter­action is entirely insignificant in the cationic component. The percentage contribution of further weak inter­actions such as O⋯C and C⋯C is negligible.

## 6. Synthesis and crystallization

NiCl_2_ (0.130 g, 1.0 mmol) was dissolved in a small amount of water. 4-Hy­droxy­benzoic acid (0.276 g, 2 mmol) was dissolved in a mixed solvent of 2 ml of absolute alcohol and 2 ml of distilled water. After dropwise addition of the PHBA solution and MEA to the nickel salt solution, the color changed gradually to light green. The resultant solution was stirred for 1 h with a magnetic stirrer at 318 K. The solution was allowed to stand at room temperature in a beaker with small holes in the cover for evaporation. About three weeks later, rectangular block-shaped single crystals of [Ni(H_2_O)_6_](PHBA)_2_(H_2_O)_2_ appeared. Analysis calculated: NiC_12_H_26_O_12_: C, 34.22%; H, 6.18%. Found: C, 33.63%; H, 6.25%.

## 7. Refinement

Crystal data, data collection and structure refinement details for the structure of compound **1** are summarized in Table 2 ▸. The hydrogen atoms of water mol­ecules and the hydroxyl group of the PHB anion were located in difference-Fourier maps and refined freely. The H atoms of the benzene ring were calculated geometrically with C—H = 0.93 Å and *U*_iso_(H) = 1.2*U*_eq_(C).

## Supplementary Material

Crystal structure: contains datablock(s) I. DOI: 10.1107/S205698902200531X/yz2020sup1.cif

Structure factors: contains datablock(s) I. DOI: 10.1107/S205698902200531X/yz2020Isup2.hkl

CCDC reference: 2131925

Additional supporting information:  crystallographic information; 3D view; checkCIF report

## Figures and Tables

**Table 1:** Hydrogen-bond geometry (Å, °)

*D*—H⋯*A*	*D*—H	H⋯*A*	*D*⋯*A*	*D*—H⋯*A*
O3—H3⋯O4	0.86 (3)	1.84 (3)	2.655 (2)	157 (3)
O4—H4*A*⋯O5^i^	0.82 (4)	1.97 (4)	2.785 (2)	173 (4)
O4—H4*B*⋯O2^ii^	0.87 (3)	1.88 (3)	2.724 (2)	163 (3)
O5—H5*A*⋯O4^iii^	0.87 (3)	1.91 (3)	2.770 (2)	169 (3)
O5—H5*B*⋯O1	0.92 (4)	1.73 (4)	2.632 (2)	168 (3)
O6—H6*A*⋯O2^iv^	0.81 (3)	1.97 (3)	2.779 (2)	174 (3)
O6—H6*B*⋯O2^v^	0.79 (4)	2.00 (4)	2.748 (2)	157 (4)
O7—H7*A*⋯O3^iii^	0.84 (3)	1.88 (3)	2.723 (2)	176 (3)
O7—H7*B*⋯O1^vi^	0.93 (4)	1.79 (4)	2.675 (2)	159 (3)

Symmetry codes: (i) 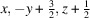 ; (ii) 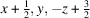 ; (iii) 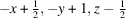 ; (iv) 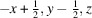 ; (v) 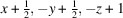 ; (vi) 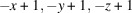 .

**Table 2:** Experimental details

Crystal data	
Chemical formula	[Ni(H_2_O)_6_](C_7_H_5_O_3_)_2_·2H_2_O
*M*_r_	477.06
Crystal system, space group	Orthorhombic, *P* *b* *c* *a*
Temperature (K)	293
*a*, *b*, *c* (Å)	11.0812 (2), 7.63258 (17), 23.7986 (5)
*V* (Å^3^)	2012.84 (7)
*Z*	4
Radiation type	Cu *K*α
μ (mm^−1^)	2.05
Crystal size (mm)	0.2 × 0.18 × 0.15

Data collection	
Diffractometer	XtaLAB Synergy, single source at home/near, HyPix3000
Absorption correction	Multi-scan (*CrysAlis PRO*; Rigaku OD, 2020 ▸)
*T*_min_, *T*_max_	0.362, 1.000
No. of measured, independent and observed [*I* > 2σ(*I*)] reflections	9865, 1949, 1656
*R*_int_	0.033
(sin θ/λ)_max_ (Å^−1^)	0.615

Refinement	
*R*[*F* ^2^ > 2σ(*F* ^2^)], *wR*(*F* ^2^), *S*	0.034, 0.101, 1.05
No. of reflections	1949
No. of parameters	169
H-atom treatment	H atoms treated by a mixture of independent and constrained refinement
Δρ_max_, Δρ_min_ (e Å^−3^)	0.25, −0.43

Computer programs: *CrysAlis PRO* (Rigaku OD, 2020 ▸), *SHELXT2018/2* (Sheldrick, 2015*a* ▸), *SHELXL2018/3* (Sheldrick, 2015*b* ▸), *Mercury* (Macrae *et al.*, 2020 ▸), and *OLEX2* (Dolomanov *et al.*, 2009 ▸).

## supplementary crystallographic information

### Crystal data

**Table:**

[Ni(H_2_O)_6_](C_7_H_5_O_3_)_2_·2H_2_O	*D*_x_ = 1.574 Mg m^−^^3^
*M**_r_* = 477.06	Cu *K*α radiation, λ = 1.54184 Å
Orthorhombic, *P**b**c**a*	Cell parameters from 4076 reflections
*a* = 11.0812 (2) Å	θ = 3.7–70.5°
*b* = 7.63258 (17) Å	µ = 2.05 mm^−^^1^
*c* = 23.7986 (5) Å	*T* = 293 K
*V* = 2012.84 (7) Å^3^	Prism, clear greenish green
*Z* = 4	0.2 × 0.18 × 0.15 mm
*F*(000) = 1000	

### Data collection

**Table:**

XtaLAB Synergy, single source at home/near, HyPix3000 diffractometer	1656 reflections with *I* > 2σ(*I*)
Detector resolution: 10.0000 pixels mm^-1^	*R*_int_ = 0.033
ω scans	θ_max_ = 71.6°, θ_min_ = 3.7°
Absorption correction: multi-scan (CrysAlisPro; Rigaku OD, 2020)	*h* = −13→13
*T*_min_ = 0.362, *T*_max_ = 1.000	*k* = −9→9
9865 measured reflections	*l* = −29→29
1949 independent reflections	

### Refinement

**Table:**

Refinement on *F*^2^	0 restraints
Least-squares matrix: full	Hydrogen site location: mixed
*R*[*F*^2^ > 2σ(*F*^2^)] = 0.034	H atoms treated by a mixture of independent and constrained refinement
*wR*(*F*^2^) = 0.101	*w* = 1/[σ^2^(*F*_o_^2^) + (0.0601*P*)^2^ + 0.4799*P*] where *P* = (*F*_o_^2^ + 2*F*_c_^2^)/3
*S* = 1.05	(Δ/σ)_max_ < 0.001
1949 reflections	Δρ_max_ = 0.25 e Å^−^^3^
169 parameters	Δρ_min_ = −0.43 e Å^−^^3^

### Special details

**Table:**

Geometry. All esds (except the esd in the dihedral angle between two l.s. planes) are estimated using the full covariance matrix. The cell esds are taken into account individually in the estimation of esds in distances, angles and torsion angles; correlations between esds in cell parameters are only used when they are defined by crystal symmetry. An approximate (isotropic) treatment of cell esds is used for estimating esds involving l.s. planes.

### Fractional atomic coordinates and isotropic or equivalent isotropic displacement parameters (Å^2^)

**Table:**

	*x*	*y*	*z*	*U*_iso_*/*U*_eq_	
Ni1	0.500000	0.500000	0.500000	0.03036 (17)	
O5	0.32084 (12)	0.5623 (2)	0.48130 (6)	0.0376 (3)	
O7	0.54536 (14)	0.5367 (2)	0.41740 (6)	0.0398 (3)	
O6	0.46162 (15)	0.2410 (2)	0.48559 (7)	0.0419 (3)	
O2	0.04307 (14)	0.5293 (2)	0.58057 (6)	0.0428 (4)	
O4	0.31243 (14)	0.6490 (2)	0.90986 (7)	0.0480 (4)	
O3	0.12531 (15)	0.5789 (3)	0.84390 (6)	0.0582 (5)	
O1	0.23544 (12)	0.6102 (2)	0.58333 (6)	0.0505 (4)	
C1	0.13366 (16)	0.5753 (3)	0.66995 (8)	0.0345 (4)	
C2	0.22791 (16)	0.6504 (3)	0.70019 (8)	0.0374 (4)	
H2	0.293016	0.698597	0.680979	0.045*	
C4	0.13027 (16)	0.5798 (3)	0.78679 (8)	0.0384 (4)	
C7	0.13809 (17)	0.5705 (3)	0.60713 (8)	0.0376 (4)	
C3	0.22651 (17)	0.6547 (3)	0.75802 (8)	0.0393 (4)	
H3A	0.289389	0.707250	0.777671	0.047*	
C6	0.03798 (18)	0.5014 (3)	0.69949 (9)	0.0386 (4)	
H6	−0.025565	0.450481	0.679870	0.046*	
C5	0.03600 (19)	0.5025 (3)	0.75756 (9)	0.0418 (5)	
H5	−0.028059	0.451858	0.776878	0.050*	
H6A	0.456 (3)	0.183 (4)	0.5140 (13)	0.057 (8)*	
H5A	0.286 (3)	0.486 (4)	0.4597 (12)	0.065 (9)*	
H7A	0.494 (3)	0.496 (3)	0.3948 (15)	0.061 (10)*	
H6B	0.499 (3)	0.183 (5)	0.4642 (15)	0.075 (10)*	
H7B	0.619 (4)	0.487 (4)	0.4080 (14)	0.081 (10)*	
H5B	0.282 (3)	0.586 (4)	0.5145 (15)	0.085 (10)*	
H4A	0.318 (3)	0.739 (5)	0.9287 (14)	0.088 (11)*	
H4B	0.385 (3)	0.609 (4)	0.9056 (13)	0.085 (10)*	
H3	0.192 (3)	0.619 (4)	0.8573 (14)	0.083 (10)*	

### Atomic displacement parameters (Å^2^)

**Table:**

	*U*^11^	*U*^22^	*U*^33^	*U*^12^	*U*^13^	*U*^23^
Ni1	0.0238 (3)	0.0401 (3)	0.0272 (3)	0.00122 (16)	0.00049 (15)	−0.00160 (16)
O5	0.0259 (6)	0.0535 (9)	0.0333 (7)	0.0017 (6)	0.0007 (5)	−0.0023 (6)
O7	0.0314 (7)	0.0587 (9)	0.0293 (7)	0.0001 (7)	0.0013 (5)	−0.0014 (6)
O6	0.0479 (8)	0.0403 (8)	0.0376 (8)	−0.0021 (7)	0.0052 (7)	−0.0043 (7)
O2	0.0392 (8)	0.0534 (9)	0.0357 (8)	0.0008 (6)	−0.0059 (6)	−0.0053 (6)
O4	0.0352 (8)	0.0584 (10)	0.0505 (9)	−0.0020 (7)	−0.0041 (6)	−0.0072 (7)
O3	0.0390 (8)	0.1039 (14)	0.0317 (7)	−0.0147 (9)	−0.0008 (6)	0.0051 (8)
O1	0.0332 (7)	0.0840 (11)	0.0343 (7)	0.0053 (7)	0.0055 (5)	−0.0011 (7)
C1	0.0287 (8)	0.0402 (10)	0.0344 (10)	0.0023 (8)	0.0004 (7)	−0.0011 (7)
C2	0.0299 (9)	0.0441 (11)	0.0383 (10)	−0.0054 (8)	0.0022 (7)	0.0008 (8)
C4	0.0309 (9)	0.0508 (11)	0.0333 (9)	0.0010 (9)	−0.0001 (7)	0.0027 (8)
C7	0.0320 (9)	0.0441 (11)	0.0365 (10)	0.0075 (8)	−0.0003 (7)	−0.0031 (8)
C3	0.0318 (9)	0.0477 (11)	0.0385 (10)	−0.0071 (8)	−0.0024 (7)	−0.0010 (8)
C6	0.0279 (9)	0.0486 (12)	0.0392 (10)	−0.0037 (8)	−0.0030 (8)	−0.0009 (8)
C5	0.0283 (9)	0.0571 (13)	0.0400 (11)	−0.0059 (8)	0.0020 (8)	0.0049 (8)

### Geometric parameters (Å, º)

**Table:**

Ni1—O5^i^	2.0893 (13)	O3—C4	1.360 (2)
Ni1—O5	2.0893 (13)	O3—H3	0.86 (4)
Ni1—O7	2.0482 (13)	O1—C7	1.256 (2)
Ni1—O7^i^	2.0483 (13)	C1—C2	1.392 (3)
Ni1—O6^i^	2.0511 (15)	C1—C7	1.496 (3)
Ni1—O6	2.0511 (15)	C1—C6	1.391 (3)
O5—H5A	0.87 (3)	C2—H2	0.9300
O5—H5B	0.92 (4)	C2—C3	1.377 (3)
O7—H7A	0.84 (3)	C4—C3	1.390 (3)
O7—H7B	0.92 (4)	C4—C5	1.387 (3)
O6—H6A	0.81 (3)	C3—H3A	0.9300
O6—H6B	0.79 (3)	C6—H6	0.9300
O2—C7	1.268 (2)	C6—C5	1.382 (3)
O4—H4A	0.82 (4)	C5—H5	0.9300
O4—H4B	0.86 (4)		
			
O5—Ni1—O5^i^	180.00 (8)	H4A—O4—H4B	107 (3)
O7—Ni1—O5^i^	90.14 (6)	C4—O3—H3	110 (2)
O7—Ni1—O5	89.86 (6)	C2—C1—C7	120.13 (17)
O7^i^—Ni1—O5	90.14 (6)	C6—C1—C2	118.52 (17)
O7^i^—Ni1—O5^i^	89.86 (6)	C6—C1—C7	121.33 (17)
O7—Ni1—O7^i^	180.0	C1—C2—H2	119.4
O7^i^—Ni1—O6^i^	91.28 (7)	C3—C2—C1	121.21 (17)
O7^i^—Ni1—O6	88.72 (7)	C3—C2—H2	119.4
O7—Ni1—O6	91.28 (7)	O3—C4—C3	121.67 (18)
O7—Ni1—O6^i^	88.72 (7)	O3—C4—C5	117.95 (17)
O6—Ni1—O5^i^	90.77 (6)	C5—C4—C3	120.37 (18)
O6—Ni1—O5	89.23 (6)	O2—C7—C1	118.49 (17)
O6^i^—Ni1—O5	90.77 (6)	O1—C7—O2	123.26 (18)
O6^i^—Ni1—O5^i^	89.23 (6)	O1—C7—C1	118.24 (17)
O6—Ni1—O6^i^	180.0	C2—C3—C4	119.41 (17)
Ni1—O5—H5A	113 (2)	C2—C3—H3A	120.3
Ni1—O5—H5B	108 (2)	C4—C3—H3A	120.3
H5A—O5—H5B	116 (3)	C1—C6—H6	119.5
Ni1—O7—H7A	114 (2)	C5—C6—C1	120.97 (18)
Ni1—O7—H7B	113 (2)	C5—C6—H6	119.5
H7A—O7—H7B	107 (3)	C4—C5—H5	120.3
Ni1—O6—H6A	114 (2)	C6—C5—C4	119.50 (18)
Ni1—O6—H6B	123 (2)	C6—C5—H5	120.3
H6A—O6—H6B	106 (4)		
			
O3—C4—C3—C2	179.3 (2)	C7—C1—C2—C3	−179.30 (18)
O3—C4—C5—C6	179.88 (19)	C7—C1—C6—C5	178.40 (18)
C1—C2—C3—C4	1.2 (3)	C3—C4—C5—C6	−0.4 (3)
C1—C6—C5—C4	0.6 (3)	C6—C1—C2—C3	−1.0 (3)
C2—C1—C7—O2	−168.06 (18)	C6—C1—C7—O2	13.7 (3)
C2—C1—C7—O1	10.9 (3)	C6—C1—C7—O1	−167.37 (19)
C2—C1—C6—C5	0.1 (3)	C5—C4—C3—C2	−0.5 (3)

Symmetry code: (i) −*x*+1, −*y*+1, −*z*+1.

### Hydrogen-bond geometry (Å, º)

**Table:**

*D*—H···*A*	*D*—H	H···*A*	*D*···*A*	*D*—H···*A*
O3—H3···O4	0.86 (3)	1.84 (3)	2.655 (2)	157 (3)
O4—H4*A*···O5^ii^	0.82 (4)	1.97 (4)	2.785 (2)	173 (4)
O4—H4*B*···O2^iii^	0.87 (3)	1.88 (3)	2.724 (2)	163 (3)
O5—H5*A*···O4^iv^	0.87 (3)	1.91 (3)	2.770 (2)	169 (3)
O5—H5*B*···O1	0.92 (4)	1.73 (4)	2.632 (2)	168 (3)
O6—H6*A*···O2^v^	0.81 (3)	1.97 (3)	2.779 (2)	174 (3)
O6—H6*B*···O2^vi^	0.79 (4)	2.00 (4)	2.748 (2)	157 (4)
O7—H7*A*···O3^iv^	0.84 (3)	1.88 (3)	2.723 (2)	176 (3)
O7—H7*B*···O1^i^	0.93 (4)	1.79 (4)	2.675 (2)	159 (3)

Symmetry codes: (i) −*x*+1, −*y*+1, −*z*+1; (ii) *x*, −*y*+3/2, *z*+1/2; (iii) *x*+1/2, *y*, −*z*+3/2; (iv) −*x*+1/2, −*y*+1, *z*−1/2; (v) −*x*+1/2, *y*−1/2, *z*; (vi) *x*+1/2, −*y*+1/2, −*z*+1.

## References

[bb1] Baruah, J. B. (2016). Private communication (refcode AYOJOP). CCDC, Cambridge, England. https://doi.org/10.5517/ccdc.csd.ccqxky3

[bb2] Bednarchuk, T. J., Kinzhybalo, V. & Pietraszko, A. (2016). *Acta Cryst.* C**72**, 432–441. 10.1107/S2053229616006458 27146574

[bb3] Bergmann, H. & Eckert, H. (1990). *Plant Growth Regul.* **9**, 1–8.

[bb4] Bruno, I. J., Cole, J. C., Edgington, P. R., Kessler, M., Macrae, C. F., McCabe, P., Pearson, J. & Taylor, R. (2002). *Acta Cryst.* B**58**, 389–397. 10.1107/s0108768102003324 12037360

[bb5] Cho, J.-Y., Moon, J.-H., Seong, K.-Y. & Park, K.-H. (1998). *Biosci. Biotechnol. Biochem.* **62**, 2273–2276. 10.1271/bbb.62.2273 9972252

[bb6] Dolomanov, O. V., Bourhis, L. J., Gildea, R. J., Howard, J. A. K. & Puschmann, H. (2009). *J. Appl. Cryst.* **42**, 339–341.

[bb7] Etter, M. C., MacDonald, J. C. & Bernstein, J. (1990). *Acta Cryst.* B**46**, 256–262. 10.1107/s0108768189012929 2344397

[bb8] Gomathi, S. & Muthiah, P. T. (2013). *Acta Cryst.* C**69**, 1498–1502. 10.1107/S0108270113031429 24311499

[bb9] Groom, C. R., Bruno, I. J., Lightfoot, M. P. & Ward, S. C. (2016). *Acta Cryst.* B**72**, 171–179. 10.1107/S2052520616003954 PMC4822653 27048719

[bb10] Hassan, F., Fayez, M. & Abdalla, N. (2020). *Open J. Inorg. Non-metallic Materials*, **10**, 15–29.

[bb11] Ibragimov, A. B., Ashurov, J. M., Ibragimov, A. B. & Zakirov, B. S. (2017*b*). *Russ. J. Inorg. Chem.* **62**, 439–445.

[bb12] Ibragimov, A. B., Ashurov, J. M., Ibragimov, B. T. & Zakirov, B. S. (2017*c*). *J. Mol. Struct.* **1128**, 307–316.

[bb13] Ibragimov, A. B., Ashurov, J. M. & Zakirov, B. S. (2017*a*). *J. Struct. Chem.* **58**, 588–590.

[bb14] Ibragimov, A. B., Englert, U., Ashurov, J. M. & Wang, A. (2018). *J. Struct. Chem.* **59**, 411–414.

[bb15] Lo, K. M., Lee, S. M. & Tiekink, E. R. T. (2020). *Z. Krist. New Cryst. Struct.* **235**, 313–315.

[bb16] Macrae, C. F., Sovago, I., Cottrell, S. J., Galek, P. T. A., McCabe, P., Pidcock, E., Platings, M., Shields, G. P., Stevens, J. S., Towler, M. & Wood, P. A. (2020). *J. Appl. Cryst.* **53**, 226–235. 10.1107/S1600576719014092 PMC6998782 32047413

[bb17] Manuja, R., Sachdeva, Sh., Jain, A. & Chaudhary, J. (2013). *Int. J. Pharm. Sci. Rev. Res*, **22**, 109–115.

[bb18] Moussa, H. R., El-Sayed, M. S. & Ghramh, H. A. (2019). *Int. J.Veg. Sci*. **18**, Article No. 185. https://doi.org/10.1186/s12934-019-1233-7

[bb19] Rigaku OD (2020). *CrysAlis PRO*. Rigaku Oxford Diffraction, Yarnton, England.

[bb20] Sekine, Y., Aliyah, K. H., Shimada, T., Zhang, J., Kosaka, W. & Miyasaka, H. (2018). *Chem. Lett.* **47**, 693–696.

[bb21] Sheldrick, G. M. (2015*a*). *Acta Cryst.* A**71**, 3–8.

[bb22] Sheldrick, G. M. (2015*b*). *Acta Cryst.* C**71**, 3–8.

[bb23] Shnulin, A. N., Nadzhafov, G. N., Amiraslanov, I. R., Usubaliev, B. T. & Mamedov, Kh. S. (1981). *Russ. J. Coord. Chem.* **7**, 1409–1417.

[bb24] Shnulin, A. N., Nadzhafov, G. N. & Mamedov, Kh. S. (1984). *J. Struct. Chem.* **25**, 421–429.

[bb25] Spackman, P. R., Turner, M. J., McKinnon, J. J., Wolff, S. K., Grimwood, D. J., Jayatilaka, D. & Spackman, M. A. (2021). *J. Appl. Cryst.* **54**, 1006–1011. 10.1107/S1600576721002910 PMC8202033 34188619

[bb26] Sytar, O., Brestic, M., Rai, M. & Shao, H.-B. (2012). *J. Med. Plants Res.* **6**, 2526–2539.

[bb27] Tran, Q. H. & Doan, T. T. (2020). *New J. Chem.* **44**, 13036–13045.

[bb28] Zardini, H. Z., Davarpanah, M., Shanbedi, M., Amiri, A., Maghrebi, M. & Ebrahimi, L. (2014). *J. Biomed. Mater. Res.* **102**, 1774–1781. 10.1002/jbm.a.34846 23784887

